# Anterior pushing technique for a broken scalpel blade in lumbar discectomy: a case report

**DOI:** 10.3389/fsurg.2023.1266102

**Published:** 2023-09-27

**Authors:** Dejan Čeleš, Mladen Gasparini, Janez Mohar

**Affiliations:** ^1^ Department of Spine Surgery, Valdoltra Orthopaedic Hospital, Ankaran, Slovenia; ^2^ Department of Surgery, General Hospital Izola, Izola, Slovenia; ^3^ Chair of Orthopaedics, Faculty of Medicine, University of Ljubljana, Ljubljana, Slovenia

**Keywords:** broken scalpel blade, discectomy, retroperitoneal space, CT angiography, laparoscopy, anterior pushing technique

## Abstract

The presence of broken surgical blades or other surgically uncontrolled sharp and pointed objects in the disc space is a rare but potentially severe complication of posterior lumbar spine procedures. Herein, we report the case of a 59-year-old female patient with a history of lumbar decompression and interspinous process device implantation who underwent an instrumented revision of the lumbosacral junction. During the L5–S1 discectomy, the scalpel blade broke, and the broken fragment could not be retrieved through the posterior approach. With regard to the vascular anatomy, we partially pushed the fragment through the anterior annulus into the retroperitoneal space. In addition, pedicle screws were locked to ensure the stability of the construct. The fractured blade fragment was eventually removed by laparoscopy 1 week after the initial procedure. This experience suggests that the anterior pushing technique with fluoroscopy is an option in rare cases where a broken scalpel blade cannot be reached through the posterior approach. In such cases, computed tomography angiography is recommended.

## 1. Introduction

Vascular injuries during lumbar spine surgery are rare, accounting for only 1–5 cases per 10,000 lumbar disc surgeries (1), and studies on managing broken blades are scarce. Furthermore, to the best of our knowledge, a management algorithm for the adverse event of anterior migration of a fractured scalpel blade has not yet been published. In the present report, we describe the case management of a broken scalpel on the L5–S1 disc in a 59-year-old female patient who underwent a laminotomy and an implantation of an interspinous process device at the L4–L5 motion segment 7 years before the L3–S1 decompression and instrumented fusion were planned as treatment. The patient was subsequently scheduled for revision surgery due to degenerative lateral spinal canal stenosis, worsening leg pain, and chronic mechanical back pain.

## 2. Case presentation

In the present case, while performing a continuous-motion oval-shaped incision of the posterior disc annulus during L5–S1 discectomy and transforaminal interbody cage implantation, the No. 15 scalpel blade broke. An attempt was made to retrieve the broken segment through the posterior disc space using a microscope under fluoroscopic control. However, visual confirmation of the fractured fragment was impossible as it was covered with disc material.

## 3. Case timeline

**Figure 1:**
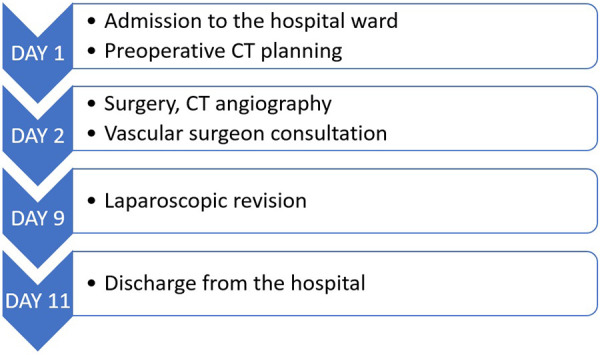
Case management timeline.

Figure 1 shows the management timeline of the presented case.

## 4. Diagnostic assessment

Intraoperatively, we reviewed the vascular anatomy of the patient using magnetic resonance (MR) imaging and preoperative computed tomography (CT). In addition, we performed an intraoperative 3D C-arm CT scan to visualize the position of the broken blade.

## 5. Therapeutic intervention

**Figure 2:**
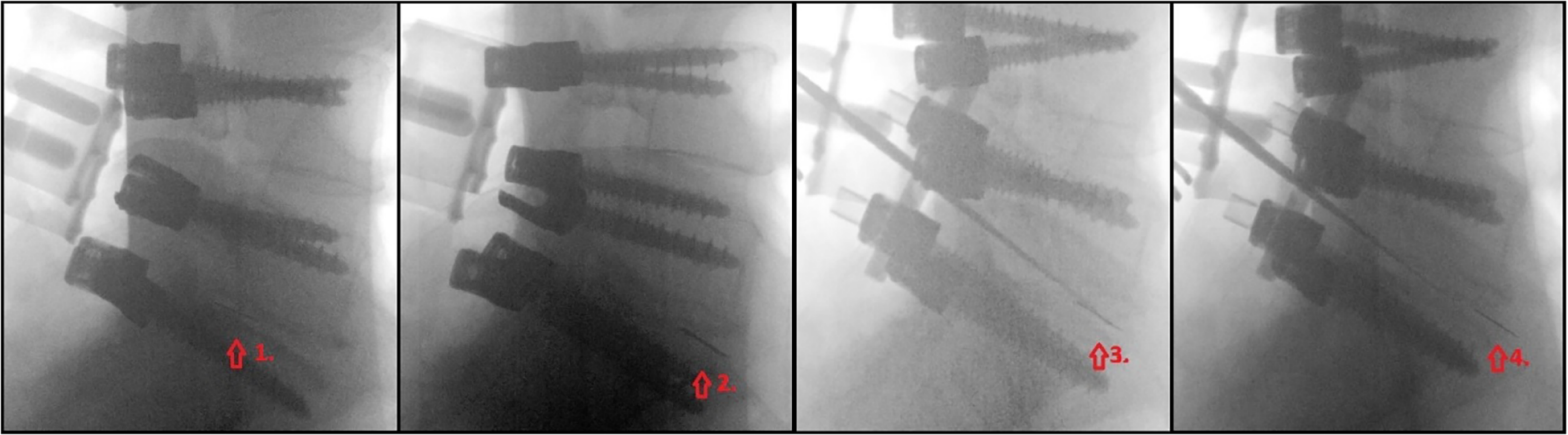
Sequential x-ray images of the anterior blade pushing technique.

Removal using a pituitary rongeur was unsuccessful, resulting in the partial anterior migration of the broken scalpel blade. When the broken blade piece was in the anterior third of the disc space, we tried to remove it using a nerve hook, which was again unsuccessful. Afterward, we reevaluated the vascular anatomy using preoperative MR and CT scans. We partially pushed the broken blade piece through the anterior annulus using the nerve hook already in the disc space, guided by serial X-ray imaging (Figure 2). After the pushing maneuver, pedicle screws were locked to the rod to ensure the stability of the construct. The patient was mobilized and resumed regular ambulation as per the standard practice.

**Figure 3:**
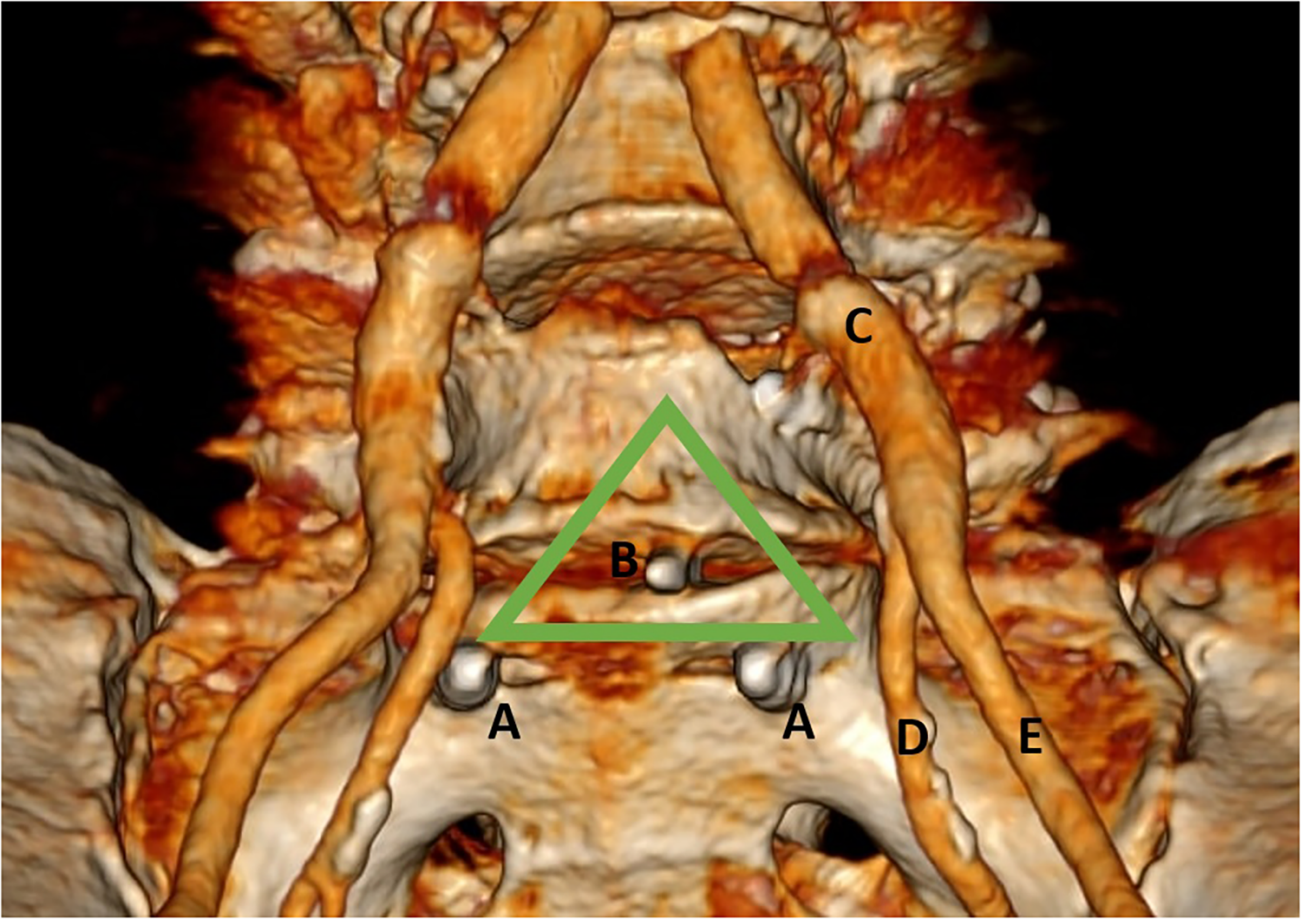
Computed tomography angiography image following three-dimensional reconstruction, demonstrating the (A) tip of the pedicle screw, (B) broken scalpel blade piece in the disc space, (C) common iliac artery, (D) internal iliac artery, and (E) external iliac artery. The green triangle indicates the proposed safe triangular corridor.

Furthermore, CT angiography was conducted before the patient was referred to a vascular surgeon (Figure 3), and laparoscopic revision was performed a week later. This procedure revealed that the omentum majus was detached from the anterior abdominal wall, and the L5 vertebral body was accessed from the medial side of the mesosigmoid colon. After applying cranial traction to the sigmoid colon, the mesocolon of the rectosigmoid became tense, and a peritoneal incision was made. Furthermore, the right iliac artery, ureter, and sacral promontory were identified. The presacral structures were then lifted using a meticulous technique, allowing for the successful removal of the broken blade piece from where it was lodged in the anterior disc annulus.

## 6. Follow-up and outcomes

The postoperative course of the patient was uneventful. At the 6-month follow-up, she was pain-free, with a stable L3–S1 fusion construct.

## 7. Discussion

In terms of anatomy, injury to the iliac artery and its branches is more likely to occur caudal to the L4 midbody. In contrast, the iliac vein and its branches may be damaged caudal to the L4–L5 disc space. Furthermore, the right internal iliac artery and vein are susceptible to injury at the L5–S1 disc level. Aortic and caval injuries most commonly occur at the L1–L4 vertebral levels (1). In addition, the right and left common iliac arteries are the most commonly injured (43% and 29%, respectively) during lumbar discectomy, whereas the right common iliac vein is the least commonly affected vessel (2).

Complications that may arise from protrusion or extrusion of a sharp object anterior to the lumbar disc space include arteriovenous fistula; pseudoaneurysm; vascular, urethral, or bowel laceration; foreign body granuloma; and intravascular migration of a foreign body (1, 3–8). The risk factors for scalpel blade fracture during discectomy include the use of No. 15 blades with a narrow junction, the presence of a calcified posterior longitudinal ligament and annulus, the application of the scalpel blade stabbing-and-turning technique, and encountering a narrowed disc space (9, 10). An anterior annulus ligament defect also increases the risk of anterior protrusion of the blade (11).

We considered laparoscopy (with or without a robotic aid) a minimally invasive, safe, and optimal method to visualize critical anatomical structures. In addition, robot-assisted procedures offer enhanced dexterity, allowing greater precision when dissecting (12–14). In the present study, we proposed a safe triangular corridor for avoiding vascular lesions (Figure 3). Depending on the vascular anatomy of a patient, a pushing maneuver is recommended for scalpel blade pieces retained at specific L5–S1 levels, as higher lumbar levels have a higher risk of vessel laceration. By employing the pushing technique, a minimally invasive anterior approach was possible for retrieving the broken scalpel blade lodged in the anterior disc annulus.

Relevant medical literature on such complications is scarce. Transforaminal, anterior-laparoscopic, posterior-endoscopic, and lateral approaches were used to retrieve broken scalpel blade fragments (3, 10, 12, 15). The limitation of this study is that case reports are rare, making each case unique.

## 8. Conclusions

Retained and extruded sharp objects within and anterior to the disc space in the lumbosacral spine are potentially life-threatening complications. These can be difficult to treat and may require an interdisciplinary approach to treatment. Vascular injury can manifest in different forms and may initially present as asymptomatic. We suggest using a stabbing technique instead of a continuous stabbing-and-turning excision of the posterior annulus to avoid breaking the scalpel blade. A safe triangular corridor should be considered for the anterior pushing technique. We suggest considering a robot-assisted laparoscopy as the preferred method for retrieving anteriorly extruded objects.

## 9. Patient perspective and informed consent

During the follow-up, the patient reported being free from pain and expressed satisfaction with our treatment. She had provided informed consent for the publication of this case. All personal information has been anonymized.

## Data Availability

The original contributions presented in the study are included in the article; further inquiries can be directed to the corresponding author.
